# Editorial: Otologic Trauma, Pathology, and Therapy

**DOI:** 10.3389/fncel.2022.900074

**Published:** 2022-04-13

**Authors:** Lukas D. Landegger, Takeshi Fujita, Taha A. Jan, Isabel Varela-Nieto

**Affiliations:** ^1^Department of Otolaryngology-Head and Neck Surgery, Vienna General Hospital, Medical University of Vienna, Vienna, Austria; ^2^Department of Otolaryngology-Head and Neck Surgery, Kobe University Graduate School of Medicine, Kobe, Japan; ^3^Department of Otolaryngology-Head and Neck Surgery, University of California, San Francisco, San Francisco, CA, United States; ^4^Institute for Biomedical Research “Alberto Sols”, Spanish National Research Council-Autonomous University of Madrid, Madrid, Spain; ^5^Rare Diseases Networking Biomedical Research Centre, CIBER, Carlos III Institute of Health, Madrid, Spain; ^6^La Paz Hospital Institute for Health Research, Madrid, Spain

**Keywords:** otology, hearing loss, cochlea, synaptopathy, regeneration, cisplatin, genetics

The current Research Topic tries to highlight some of the most relevant recent advances regarding sensorineural hearing loss (SNHL). The etiology of SNHL primarily involves hereditary factors, ototoxic drugs, noise, and aging, all of which trigger two main mechanisms: damage to the organ of Corti, namely mechanosensory hair cells (HCs) and non-sensory supporting cells (SCs), and/or loss of spiral ganglion neurons (SGNs) that subsequently form the auditory nerve and hence connect the inner ear to the brain.

Hereditary hearing loss accounts for one in 500 new births (Sheffield and Smith, 2019) with a range of phenotypes from profound SNHL to mild hearing loss. A variety of different tools exist to probe genetic underpinnings of hereditary hearing loss. In the article “Navigating Hereditary Hearing Loss: Pathology of the Inner Ear,” Nicolson reviews peripheral forms of hereditary hearing loss and strategies to study underlying molecular mechanisms using animal models and cell lines. Hereditary hearing loss affects different sites within the inner ear, including the stria vascularis, organ of Corti, and SGNs, all with complementary animal models for studying specific genetic defects. In humans with hearing loss, diagnostic genetic testing leads to novel variants that may have clinical implications. In their article “A Novel Variant in the TBC1D24 Lipid-Binding Pocket Causes Autosomal Dominant Hearing Loss: Evidence for a Genotype-Phenotype Correlation,” Parzefall et al. describe a novel variant of *TBC1D24* that causes autosomal dominant hearing loss. This genotype-phenotype correlation study hypothesizes that specific residues affected in TBC1D24 regulate synaptic vesicle trafficking that may be involved in molecular mechanism of disease. These variants can be studied in the future in an animal model or *in vitro* to further dissect molecular mechanisms.

The above-mentioned synapses represent the connections between (inner) HCs and SGNs. As discovered in recent years, they represent one of the most vulnerable parts of the auditory periphery with cochlear synaptopathy interrupting the communication between these two cell types, resulting in SNHL. Several strategies to prevent or mitigate this condition are presented in the current Research Topic. In their paper “Regenerative Effect of a ROCK Inhibitor, Y-27632, on Excitotoxic Trauma in an Organotypic Culture of the Cochlea,” Koizumi et al. used a model of peripheral axonal damage in the SGNs of an explant culture of mouse cochleae. They showed that Rho-associated coiled-coil containing protein kinase (ROCK) inhibitor Y-27632 regenerated SGN axons and synapses between inner HCs and the auditory nerve. In a similar *in vitro* model, Gao et al. show in the article “Insulin-Like Growth Factor 1 on the Maintenance of Ribbon Synapses in Mouse Cochlear Explant Cultures” that insulin-like growth factor 1 (IGF-1) is crucial to maintain such synapses, which is corroborated by the fact that its deficiency is a cause of human hearing loss (García-Mato et al., 2021). Furthermore, following extensive studies on the role of neurotrophins during inner ear development and, hopefully, regeneration, Kempfle et al. report in “A Novel Small Molecule Neurotrophin-3 Analogue Promotes Inner Ear Neurite Outgrowth and Synaptogenesis *in vitro*” that an analogue of the neurotrophic factor neurotrophin-3 (NT-3) linked to bone-binding bisphosphonates also promotes synaptogenesis *in vitro*. These studies further open the possibility to preserve ribbon synapses and auditory neurons even in the absence of healthy HCs, thus increasing the panel of potential novel co-treatments to be used during cochlear implantation (Lenarz, 2018). In the translational study “Spike Generators and Cell Signaling in the Human Auditory Nerve: An Ultrastructural, Super-Resolution, and Gene Hybridization Study,” Liu et al. describe high-resolution images of the human auditory nerve using transmission electron microscopy, super-resolution structured illumination microscopy, and single molecule *in situ* hybridization (*RNAscope*^®^). This investigation presents evidence for initial spike generators that are located just beneath the inner HCs. Moreover, the authors illustrate the feasibility of obtaining high quality human inner ear tissue during select procedures, demonstrating the potential of collaborative work between surgeons and scientists to advance the auditory field.

Among the intracellular events that have been repeatedly described as a cause of injury and death in the cochlea we find oxidative stress, either associated to aging or noxious stimuli, such as noise or ototoxic drugs. This Research Topic includes several articles exploring these mechanisms. Varela-Nieto et al. review the scarce data available on the role of small molecules, nitrones, with the property of being radical oxygen and nitrogen species scavengers in the article “Use of Radical Oxygen Species Scavenger Nitrones to Treat Oxidative Stress-Mediated Hearing Loss: State of the Art and Challenges.” Further, dealing with the potential of antioxidants and vasodilators to treat the consequences of noise exposure, “Antioxidants and Vasodilators for the Treatment of Noise-Induced Hearing Loss: Are They Really Effective?” by Alvarado et al. discusses the pros and cons of these therapies, as well as their potential to be translated to clinical practice.

One of the above-mentioned ototoxic drugs is the commonly used chemotherapeutic cisplatin. In human patients and animal experiments, it is usually delivered systemically. Yet, this triggers substantial morbidity in rodents, even leading to premature death. Nacher-Soler et al. describe an alternative method in “Local Cisplatin Delivery in Mouse Reliably Models Sensorineural Ototoxicity Without Systemic Adverse Effects,” where they apply a cisplatin solution into the murine middle ear, the so-called otic bulla. This model reliably resulted in the desired hearing loss and HC damage without any distress or morbidity, hence allowing the study of ototoxic effects without having to continuously worry about animal safety.

Age-related hearing loss (ARHL) is the most common form of SNHL. In the study “Protective Effects of N^1^-Methylnicotinamide Against High-Fat Diet- and Age-Induced Hearing Loss *via* Moderate Overexpression of Sirtuin 1 Protein,” Miwa showed that mice fed a high-fat diet exhibited ARHL and decreased SIRT1 and SIRT3 expression levels in the cochlea. He also showed that N^1^-methylnicotinamide (MNAM) supplementation increased SIRT1 and SIRT3 expression levels in the cochlea and suppressed ARHL. Furthermore, Altschuler et al. showed in their study, “Rapamycin Added to Diet in Late Mid-Life Delays Age-Related Hearing Loss in UMHET4 Mice,” that adding rapamycin to the diets of mice at 14 months of age delayed ARHL. These findings all indicate potential treatment strategies for SNHL.

As the inner ear is a non-regenerative organ in adult mammals, many groups try to establish the replacement of HCs as a potential therapeutic option for SNHL. A variety of strategies have been described in the literature to achieve this, with a focus on species that are able to regenerate their sensory epithelium like birds or fish, or neonatal mammalian tissues that appear more plastic. In their review, “Transcription Factor Reprogramming in the Inner Ear: Turning on Cell Fate Switches to Regenerate Sensory Hair Cells,” Iyer and Groves summarize the current state of targeted reprogramming of SCs to HCs. Specifically, they emphasize that the single transcription factor, ATOH1, is insufficient alone in adult inner ears for reprogramming. The field is coalescing on the idea that a combination of reprogramming factors will unlock the regenerative capacity of cochlear SCs in adult mouse cochleae. This presents an exciting and novel frontier in driving HC regeneration in adult mammals, a feat previously thought to be herculean.

As mentioned, the HCs of fish lateral line neuromasts are very similar to those in the inner ear, but regenerate as opposed to their mammalian counterparts. Warchol et al. used larval zebrafish to assess the response of macrophages to neomycin ototoxicity in “Macrophages Respond Rapidly to Ototoxic Injury of Lateral Line Hair Cells but Are Not Required for Hair Cell Regeneration.” The early inflammatory response was triggered by “local” macrophages and the migration of these cells was significantly lower when Src-family kinases were inhibited. Furthermore, macrophages appear not to be essential for HC regeneration in this model. Similar to zebrafish, neonatal mammalian cochlear SCs and avian basilar papilla SCs have the capacity for HC regeneration. In their article “Initiation of Supporting Cell Activation for Hair Cell Regeneration in the Avian Auditory Epithelium: An Explant Culture Model,” Matsunaga et al. focused on the avian auditory epithelium as a tool for investigating HC regeneration, establishing an explant culture model in chick basilar papillae. This model can be used to explore the molecular mechanisms of SC activation leading to HC regeneration in chick basilar papillae and the development of therapeutics for HC regeneration in the mammalian cochlea. Another regenerative strategy that has been at the forefront of hearing research for years focuses on stem/progenitor cells. In the article “Successful Treatment of Noise-Induced Hearing Loss by Mesenchymal Stromal Cells: An RNAseq Analysis of Protective/Repair Pathways,” Warnecke et al. carried out elaborate RNAseq experiments and demonstrated that mesenchymal stromal cells (MSCs) could mitigate damage sustained during severe sound trauma. Following noise exposure, mice received intracochlear injections to deliver these multipotent cells derived from human umbilical cord Wharton's jelly into perilymph and the effects on various molecular pathways were analyzed. Such studies of transplanted cells of different lineages while enticing, remain in their infancy.

In summary, this Research Topic includes a plethora of promising approaches that will hopefully result in translational clinical studies and transformative therapies benefitting patients suffering from SNHL.

## Author Contributions

All authors listed have made a substantial, direct, and intellectual contribution to the work and approved it for publication.

## Funding

TJ was supported by the NIH/NIDCD (K08DC019683). IV-N was supported by PID2020-115274RB-I00 from the Spanish MCIN/AEI/10.13039/501100011033 and FEDER.

## Conflict of Interest

The authors declare that the research was conducted in the absence of any commercial or financial relationships that could be construed as a potential conflict of interest.

## Publisher's Note

All claims expressed in this article are solely those of the authors and do not necessarily represent those of their affiliated organizations, or those of the publisher, the editors and the reviewers. Any product that may be evaluated in this article, or claim that may be made by its manufacturer, is not guaranteed or endorsed by the publisher.
